# Towards personalized and lifestyle-inspired soil management: lessons from the gut microbiome

**DOI:** 10.1038/s44485-026-00006-x

**Published:** 2026-10-30

**Authors:** Abraham L. van Eijnatten, Giulia Vultaggio, Merlijn Schram, David Cajas Muñoz, Luís Felipe Guandalin Zagatto, Elly Morriën, Paul L. E. Bodelier, Basten L. Snoek, Justine Lejoly, Wim H. van der Putten, G. F. Ciska Veen

**Affiliations:** 1 Theoretical Biology and Bioinformatics, Science4Life, Utrecht University, Utrecht, The Netherlands; 2 Department of Terrestrial Ecology, Netherlands Institute of Ecology (NIOO-KNAW), Wageningen, The Netherlands; 3 Laboratory of Nematology, Wageningen University & Research, Wageningen, The Netherlands; 4 Department of Ecosystem and Landscape Dynamics, Institute for Biodiversity and Ecosystem Dynamics, University of Amsterdam, Amsterdam, The Netherlands; 5 Department of Microbial Ecology, Netherlands Institute of Ecology (NIOO-KNAW), Wageningen, The Netherlands; 6 Department of Ecology, Radboud Institute of Biological and Environmental Sciences (RIBES), Radboud University, Nijmegen, The Netherlands

**Keywords:** Ecology, Ecology, Environmental sciences, Microbiology

## Abstract

Soil microorganisms are key drivers of ecosystem functions supporting biodiversity, food production, and climate regulation. Restoring multiple ecosystem functions under intensive land-use and climate change requires steering microbiome composition. However, this remains challenging due to the high complexity of soils and the assembly rules of their microbiomes. As the extensively studied human gut microbiome has considerable similarities with the soil microbiome, we explore advances in steering the human gut microbiome composition to improve the steering of soil microbiomes.

## Introduction

**Fig. 1:**
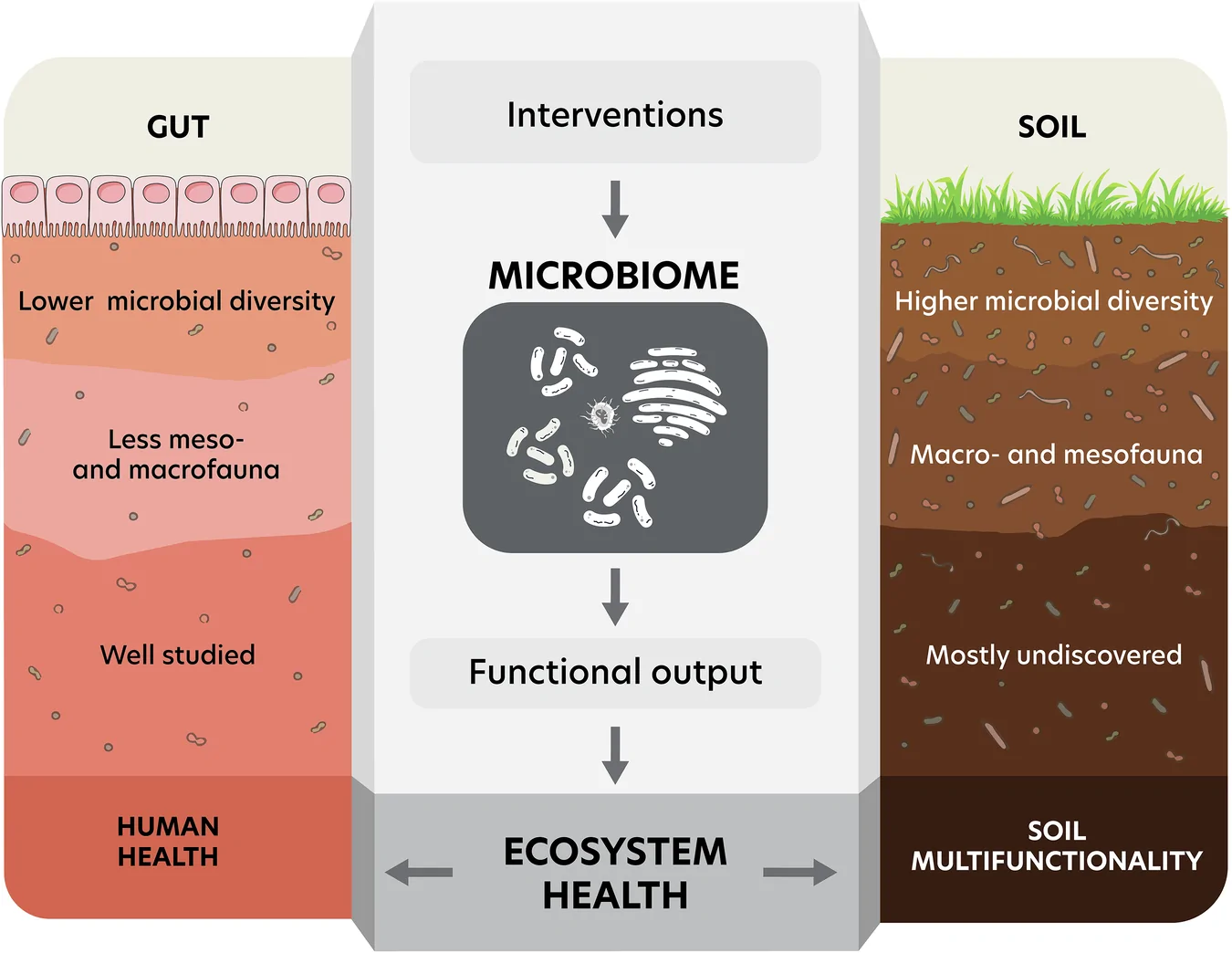
Comparison between microbiome steering in the soil and gut ecosystems. In both the gut and soil, interventions can be used to steer the microbiome towards improved functional output to enhance human health or soil multifunctionality, respectively. The gut ecosystem is simpler due to lower microbial diversity, fewer trophic levels, and more comprehensive databases. Created by Luis Felipe Guandalin Zagatto using Adobe Illustrator, incorporating Canva stock icons used under a Canva Pro license.

Soils are vital for delivering important ecosystem services such as primary productivity, climate change mitigation, disease suppression, and water and nutrient cycling^1^. Most soil functions underlying these services are carried out by soil microbes, which are at the foundation of an entire soil food web that also contains small and larger invertebrates^2^. Soil multifunctionality is defined as the soil’s capacity to perform multiple soil functions simultaneously^3–5^. Specifically, soils should be able to perform their primary function (e.g., supporting crop yield) at a high level while also performing well at climate- and environmental regulation functions (e.g., carbon storage, water purification). Trade-offs can exist between the optimization of different soil functions^1^. Soil multifunctionality strongly depends on soil microbes^6^, while ongoing agricultural intensification aimed at increasing yield is changing the biodiversity of soils^7^. Consequently, agricultural intensification can reduce soil multifunctionality by altering soil biodiversity^8,9^. Achieving a sustainable future requires understanding how to enhance soil multifunctionality while producing sufficient food for a growing world population. We propose that progress could be made by learning from current developments in human gut microbiology, where there are parallel efforts to steer the microbiome to improve (multi)functionality^10^, but knowledge and methods for microbiome steering are more advanced (Fig. 1).

In soils, the relationship between biodiversity, management, and multifunctionality still contains many unknowns. Important bottlenecks in developing this knowledge are the substantial heterogeneity of the soil substrate and microbiome as well as the high microbial diversity of soils^11^. Another barrier is that many soil microbes cannot be cultured in isolation or cannot be grown effectively on conventional media, while culturing multi-species consortia has received relatively little attention. This has hampered understanding of microbe-microbe interactions in the soil and the ability to test specific hypotheses on the relationship between microbiome taxonomy and function^12^. In gut research, high interest in human health combined with lower faunal and microbial diversity^13,14^ has resulted in a more complete understanding of the composition and function of the microbiome. Therefore, methods for steering soil microbiomes and understanding of their underlying mechanisms remain less developed than in gut microbiology. This presents an interesting learning opportunity because the gut and soil systems share parallel microbiome functions (e.g., nutrient cycling and disease suppression), as well as similar microbiome steering mechanisms such as inputs (diet/amendments) and behaviors (lifestyle/management). Building on these parallels, we aim to inspire soil studies aimed at harnessing the potential of the microbiome using concepts and methods from gut microbiology. First, we discuss how the parallel between lifestyle and management highlights the importance of consistent long-term practices. Then we explore how synbiotics can improve the effectiveness of microbial inoculants, and how phage-based interventions could be applied to steer soil microbiome composition and function. Finally, we outline how to effectively implement field-specific soil management using machine learning and metabolic modeling approaches inspired by personalized medicine.

## Long-term microbiome steering requires persistent management

Agricultural management is a powerful tool to steer soil microbiomes because management practices can have enduring effects on soil microbiomes and their functionality^15,16^. In the current agricultural system, intensive management practices such as mineral fertilization, chemical pesticide application, and tillage are routinely used to maximize primary productivity. These practices often result in concomitant decreases in the taxonomic and functional diversity of soil microbes^7,17^ and can come at the expense of soil multifunctionality^18^. Alternatively, management interventions (e.g., amendment application or crop diversification) could be intentionally designed to support soil microbiomes and soil multifunctionality^19^. For example, shifting to more extensive management (e.g., organic farming) can improve soil biodiversity and soil functioning without compromising yield^18^. While it is well established that long-term agricultural management shapes the microbiome^7,20^, it remains largely unknown how consistently such interventions must be applied for their effects on the soil microbiome to become persistent^21,22^. In contrast to soils, where the importance of consistent management has not been made explicit enough in the literature, gut microbiologists have emphasized consistency of interventions as key for persistently steering gut microbiomes^23^. Here, it is well known that consistently repeated human behaviors such as diet and exercise, collectively referred to as lifestyle, shape the microbiome of the gut^23–25^, and lifestyle interventions are therefore routinely applied to steer gut microbiomes.

While both human lifestyle and soil management can affect the resident microbiome by changing resource availability, or more generally the habitat, the transient nature of the gut substrate makes the need for behavioral consistency more intuitive. Gut microbes rely entirely on current inputs from the host’s diet, making dietary interventions a quick and effective way to steer gut microbiomes^26^. However, when the usual dietary regime is resumed, effects may be immediately reversed^27,28^. In soils, rhizosphere microbiome dynamics are similar to gut dynamics, with labile exudates from plants rapidly reshaping bacterial and fungal communities^29^.

In contrast to the gut system, soils are also affected by management practices that modify the habitat, with potentially very long-lasting effects on the soil microbiome. For example, the residence time of organic substrates applied to soil can span from days to millennia^30^ depending on their chemical composition and degree of stabilization^31^. This drives a slow release of nutrients over the entire period of substrate decomposition, shaping the composition and function of the bulk soil microbiome. Such changes to the bulk soil microbiome can also impact crop performance, since the bulk soil microbiome serves as a reservoir from which crops recruit beneficial microbes into the rhizosphere using root exudates^32^ and from which pathogens can invade the rhizosphere^33^. Organic substrates also impact soil structure, providing microhabitats that allow microbes to persist over time^11^. There has been relatively little research investigating how long effects of agricultural management interventions on soil microbiomes and soil multifunctionality last, yet initial evidence shows that these can dissipate over time^22,34–36^. For instance, 29 years after stopping manure application, the soil prokaryotic and fungal microbiome composition was found to be similar to soils that never received manure^22^. Hence, even if one-time interventions can have strong and immediate effects, microbiome composition and function in the long run are likely determined by consistency in agricultural management^23^. In soils, this means that improving multifunctionality requires a long-term commitment to beneficial management practices, a “healthy soil lifestyle”, rather than isolated interventions.

## Synbiotics for effective soil inoculations

Next to consistent management, microbial inoculations can be used to enhance soil multifunctionality. Microbial inoculants consist of selected microorganisms that are added to soil to enhance soil functions, crop performance, or overall biodiversity. Some soil inoculants consist of specific beneficial microbes^37,38^ such as rhizobia to promote nitrogen-fixing nodules in legumes^39^ and arbuscular mycorrhizal fungi to promote nutrient uptake^40^. Alternatively, soil inoculants can consist of microbial consortia or even entire microbiomes in the case of soil transplantations^41–43^. In gut microbiology, inoculations with beneficial microbial strains or consortia (probiotics) or entire microbiomes (fecal transplants) are commonly used to steer gut microbiomes. These interventions can improve gut health, for example in the case of gastrointestinal and metabolic diseases^44^. While microbial inoculations are popular tools for shifting the microbiome, the effect of inoculation can be unpredictable as inoculants are sometimes unable to establish (engraft) in an already existing microbiome^45,46^. In gut microbiology, a potential solution to unreliable engraftment is to give the targeted microbes a competitive advantage. Here, we explore how the novel concept of synbiotics can be used to provide a competitive advantage to microbial inoculants, and for targeted steering of the microbiome.

Newly inoculated microbes merge with a complex pre-existing resident microbiome that has already adapted to its environment. However, microbial community assembly is affected by the order in which species arrive, a phenomenon called “priority effects”^47^. These priority effects arise from the pre-existing community preempting resources and niches, which can inhibit the establishment of inoculants^45,46,48–51^. Such establishment failures are known in both gut^52,53^ and soil^54,55^ environments. It follows that the key to improving the success of microbial inoculations is to enhance the competitive ability of inoculated microbes.

**Fig. 2:**
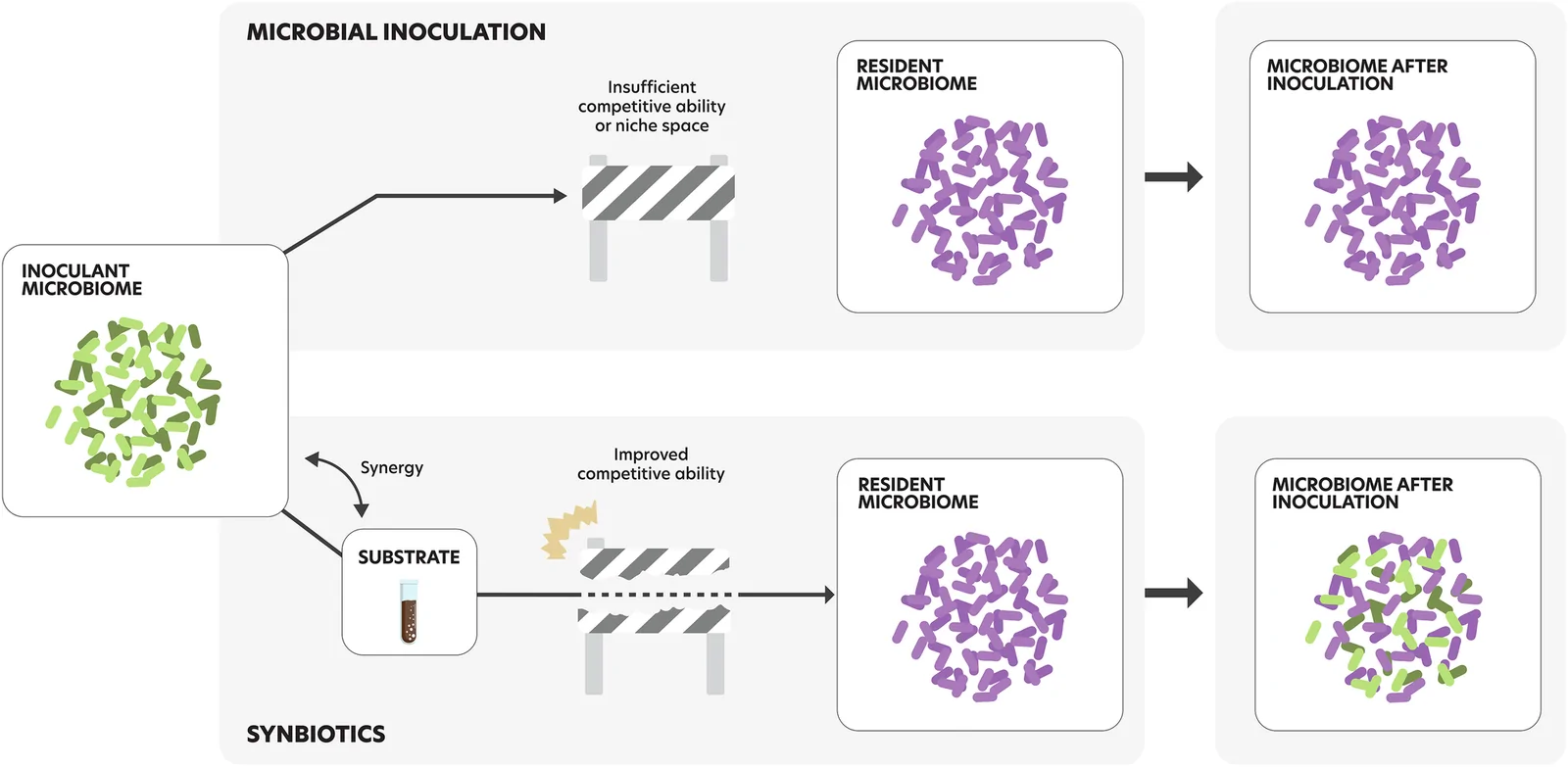
Illustration describing how synbiotics can improve the establishment of microbial inoculants. The top panel shows how inoculation can fail due to competition for niche space. The bottom panel shows how synbiotics can facilitate inoculant establishment. Created by Luis Felipe Guandalin Zagatto using Adobe Illustrator, incorporating Canva stock icons used under a Canva Pro license.

A well-established method from gut medicine to overcome these engraftment issues is to provide a synergistic combination of probiotics and substrates, with the substrates preferentially enhancing the growth of the added beneficial microbes. Inoculant products containing such combinations of microbes and preferential growth substrates are called synbiotics^56^. In synbiotics, the resource niche for the inoculant is provided along with the inoculant itself, increasing the chances of establishment^57^ (Fig. 2). For example, a now widely available synbiotic consisting of the probiotic *Bifidobacterium longum* and a preferential growth substrate (inulin oligofructose) was shown to reduce inflammation in patients with Inflammatory Bowel Disease (IBD)^58^. The synbiotic concept also shows promise in initial laboratory experiments in soils. For instance, inoculated *Pseudomonas fluorescens* failed to establish in a soil-derived natural community due to limited niche availability. This establishment failure was reversed when the inoculant was supplied with the preferential growth substrate toluene, giving it a competitive edge^59^. Other studies show benefits of combining inoculants with organic manure^60^, fertilizer^61^, or amendments^62^.

Implementing soil synbiotics requires the identification of effective combinations of microbial strains and substrates. In gut microbiology, appropriate growth substrates are found by administering a range of substrates to either bacterial consortia in the lab or patients’ guts and identifying enriched strains of the target species^63^. Promising combinations of microbes and substrates are then tested for synergistic benefits upon inoculation^63^. Alternatively, potential candidates of probiotic strains may be selected for further testing by growing them in isolation on one or more potential substrates^64^. Soil synbiotics could similarly be developed by matching specific microbial strains with substrates or compounds in vitro, or by enrichment experiments in fields or mesocosms^65^. However, the soil habitat is more intricate than the gut habitat in terms of spatial and physicochemical heterogeneity, trophic complexity, and microbial diversity. Therefore, it may be preferable to match substrates to complex communities with complementary metabolisms, mutualistic interactions, and functional redundancy. A recent study by Aguiar et al.^66^ used this approach to amplify vineyard rhizosphere communities on winemaking byproducts (ex situ enrichment on site‑relevant substrates) and selected inoculants based on enzyme activities. These lab-cultivated consortia efficiently transferred enzyme functions from donor to recipient vineyard soils. These findings show that microbial consortia can be derived from a target soil, pre-adapted to a growth substrate, and selected based on the desired function. This approach avoids the need to find a single compatible inoculant, instead enabling selection of the community with the highest functional performance on the substrate. Identifying such synbiotic combinations and developing these into routine interventions will improve the effectiveness of inoculant-based steering of soil multifunctionality.

## Phage-based interventions for microbiome steering

Another strategy for microbiome steering that is being explored in gut microbiology is the use of phages. Due to their ubiquitous nature, phages fulfill important roles in shaping soil bacterial communities through antagonistic and mutualistic interactions^67,68^. Lytic phages replicate inside susceptible bacterial hosts and then kill them, reducing the abundance of their host bacteria and allowing competitors to proliferate. Conversely, lysogenic phages integrate into host genomes and replicate passively within them, potentially altering host traits without causing cell death. Some lysogenic phages carry Auxiliary Metabolic Genes (AMGs) and integrate these into the bacterial genome, altering host metabolism to benefit both host and phage^67,69^. The ability of phage–bacteria interactions to steer microbiome composition and function makes them a promising avenue for targeted steering of soil microbiome functions^68,70^. The application of phages to target specific pathogens (phage therapy) has been studied in the human gut and soils^71,72^. Novel applications of phages beyond pathogen control include modulation of community composition and delivery of beneficial functions via AMGs.

One innovative but highly speculative phage-based application in gut microbiology is phage rehabilitation. Phage rehabilitation differs from phage therapy in aim and scope. Where phage therapy uses lytic phages to target and eliminate specific bacterial pathogens, phage rehabilitation uses lytic phages to induce community-level shifts in microbiome composition and function^73^. A potential application is the targeted suppression of specific dominant taxa, permitting the microbiome to converge to an alternative steady state with different functional outputs. This approach appears to hold promise for patients with complex diseases that involve a disbalance of the whole microbiome (dysbiosis). Examples include irritable bowel syndrome and liver disease^74,75^. Phage rehabilitation in soils, termed “ecological phage therapy,” may also hold promise for steering microbiomes. Ecological phage therapy has been effectively used to decrease the abundance of dominant soil bacterial taxa^76^ and to shift the soil bacterial community to harbor fewer antibiotic resistance genes^77^. Through its potential for steering the microbiome, phage rehabilitation can improve the success of microbial inoculants by freeing up niche space and thereby improving their establishment. This principle has been demonstrated in the human gut, where probiotic efficacy increased when it was combined with a phage cocktail targeting *E. coli*^78^. However, initial research found that priming soil with phages did not improve inoculation success^76^. Further studies are needed to explore how harmonious combinations of phages and inoculants can be developed for soils.

Another possible application of phages involves using lysogenic phages to transfer genes encoding desired functions to their bacterial hosts to directly change microbiome function. Studies in mice have shown that lysogenic phages can alter gut microbiome function^79,80^. For example, lysogenic phages were used to deliver a bacterial gene encoding an inhibitor of a pro-inflammatory enzyme to the mouse gut. This reduced enzyme activity and inflammation, pointing to potential therapies for disorders such as IBD^80^. In soil, a pilot study showed that co-inoculation of a thermophilic bacterium and its phage in composting microcosms accelerated humification. This effect came from community restructuring and augmenting metabolic capacity with AMGs, highlighting the potential of phages to steer soil microbiome function^81^.

Research into ecological phage applications is still in its initial stages, and some potential barriers need to be addressed. For example, phage rehabilitation may cause bacterial population shifts, but if soil conditions are otherwise unchanged, microbiome composition may shift back to the previous state. Furthermore, soil bacteria are immensely diverse compared to gut bacteria, while most phages have a narrow host range. This means that related taxa may share similar traits without being susceptible to the same phages, and therefore a major challenge will be identifying suitable phages for the target bacteria. Importantly, commercial application of phages in soils will require more research into their impact on the non-target microbiome^82,83^. Despite these challenges, the recent successes of phage-based interventions in the human gut suggest that they could also be a powerful tool to steer soils towards greater multifunctionality.

## Data science for “personalized soil medicine”

**Fig. 3:**
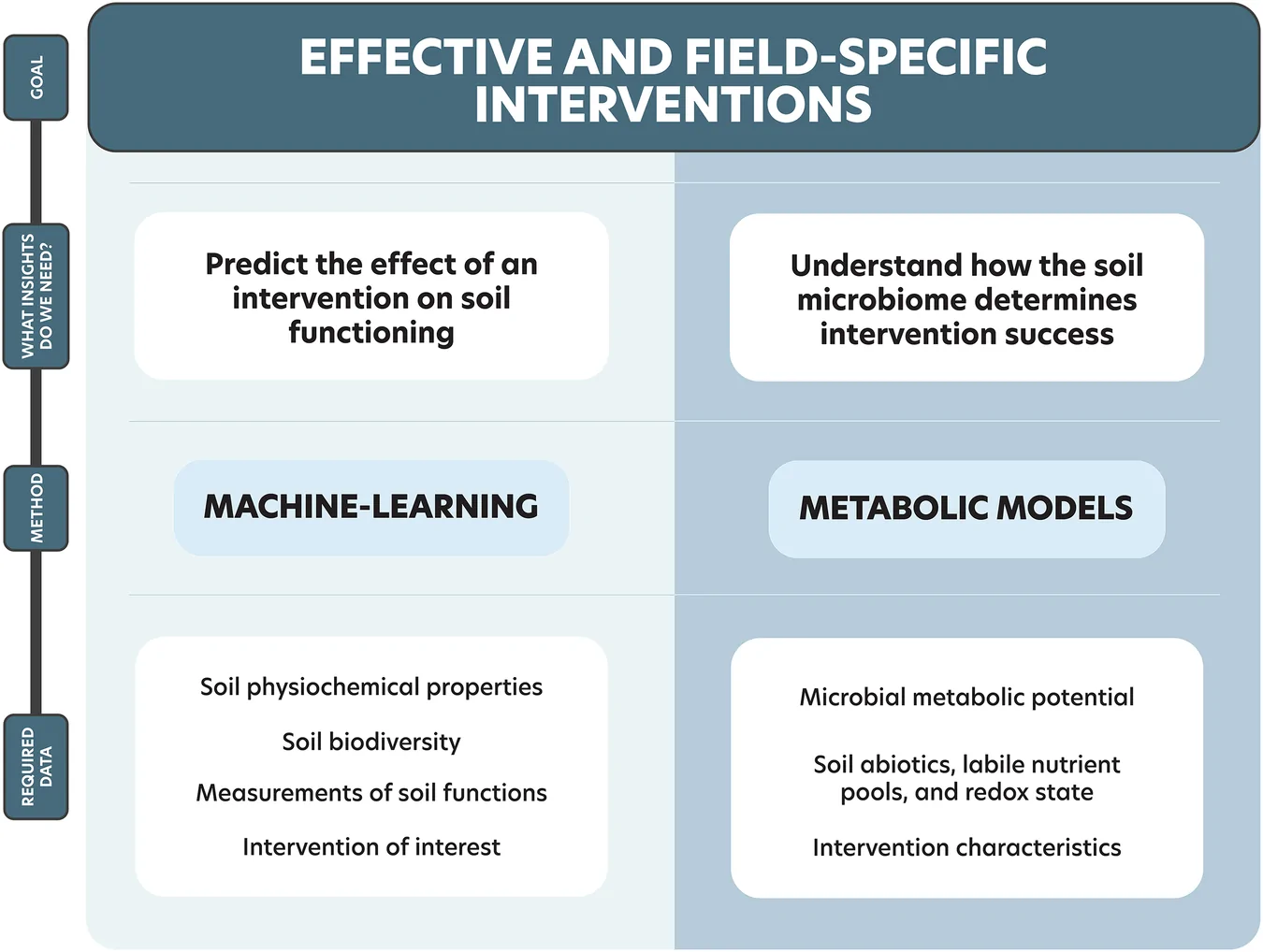
Schematic illustration of computational approaches to obtain field-specific interventions. The figure illustrates the applications of machine learning models (left panel) and metabolic models (right panel), highlighting their respective purposes and the data required for each approach. Created by Luis Felipe Guandalin Zagatto using Adobe Illustrator under a Canva Pro license.

The success of microbial interventions in soil often depends on the environmental, physicochemical, and biological context in which they are applied^84^. Therefore, steering the microbiome requires the ability to predict from context whether and to what extent an intervention will be effective in a given soil, and to understand the mechanisms that determine intervention success. However, due to the complexity of the soil, we are only at the beginning of developing such predictions and mechanistic insights. The success of interventions in medicine is similarly dependent on context. For example, the blood-sugar response to diet can be predicted from the gut microbiome, enabling the design of personalized diets^85^. It is broadly recognized that the effect of medical interventions can be improved by tailoring them to patients’ characteristics and microbiome^86–88^. This concept, called personalized medicine, relies on computational approaches such as machine learning and metabolic modeling that link individual properties (e.g., clinical data, behavior, microbiome) to the outcomes of health interventions^89^. Here, we discuss how a personalized approach to soil management can help us understand the context dependency of microbiome-based soil interventions and develop field-specific soil management (Fig. 3).

Soils are often classified into soil types based on physicochemical properties^90–92^. It is recognized that soil types may affect the success of soil interventions such as inoculations^93^, fertilization, and tillage^94^. However, biological properties are usually not considered in these classifications, whereas they may also determine the establishment success of soil inoculants. In gut medicine, patient classifications are explicitly based on the taxonomic composition of microbiomes. The gut microbiome has been classified into three “enterotypes”, each with distinct responses to dietary or probiotic interventions^95^. Furthermore, machine learning predictions of intervention outcomes based on the patient’s microbiome have proven successful, emphasizing the relationship between the gut microbiome and the response to intervention^85,96^. In addition to taxonomic composition, functional characteristics of microbiomes involving genes, gene expression, proteins, or metabolites can also be used for personalized medicine. For instance, gut metabolite levels can indicate vacant metabolic niches and predict successful inoculation of beneficial microbial strains^97,98^.

Machine learning also holds great promise for field-level predictions of intervention success. However, the abiotic drivers of the microbiome (e.g., pH, texture, moisture, nutrients) vary far more among soils than among human guts, which maintain tight homeostasis. To address this additional variation, predictive models for soils will need either very large and diverse training data, or soil type-specific training. While currently the taxonomic and functional diversity of soils is far undersampled compared to that of the human gut, extensive microbiome- and physicochemical profiling in a range of soils may bring us closer to understanding and being able to predict the context-dependency of soil interventions. Specifically, we propose measuring soil physicochemical properties, biodiversity, and functions before and after an intervention. Machine-learning models trained on these measurements will be able to learn how soil properties and biodiversity drive functional responses to interventions^99^ and to predict intervention outcomes in novel soils.

While machine learning models often identify associations without providing mechanistic understanding, metabolic models have proven to be a powerful tool in gut microbiology to understand the mechanisms behind the context dependency of interventions (Fig. 3). Metabolic models explicitly simulate the metabolic reactions taking place inside microbial cells based on gene content, yielding predictions of microbial growth rate and metabolic output in different environments. Recently, methodological advances have also enabled modeling the metabolism of gut microbiomes as a whole, capturing microbial interactions such as cross-feeding or resource competition^100–102^. For example, a recent study modeled aging mouse gut microbiomes and found that aging reduced beneficial interactions between microbes and increased the production of specific inflammatory metabolites, providing concrete therapeutic targets for aging-induced inflammation^103^. Metabolic modeling has also been used to understand how system-level output is altered by gut health interventions such as diet or probiotics and how this depends on the context of the resident microbiome. This was demonstrated by a recent study that modeled *C. difficile* infection and revealed that the mechanism behind an effective probiotic intervention was competition for specific metabolites^104^. Several additional studies have leveraged the mechanistic understanding provided by metabolic models to design personalized interventions such as probiotics, drug therapies, and diets^104–107^. For gut microbes, there are now comprehensive genome databases^108^ and databases of precomputed metabolic models^109^ to facilitate modeling complex microbiomes.

In contrast to gut microbiology, many soil microbes still lack reference genomes or precomputed metabolic models^110^. Although metagenomic data are accumulating rapidly and being integrated in soil genome databases^111–116^, more data are needed^110,111^. These expanded data combined with methodological improvements such as integration of long-read data^117^, better assembly pipelines^118–122^, ultra-deep sequencing and co-assembly^123^, and single-cell droplet barcoding approaches^124,125^ will enable the recovery of high-quality genomes at scale and their incorporation into accessible genome and metabolic model databases. An additional barrier in the implementation of soil metabolic models is the much higher fraction of dormant microbes in soils (~80–95%) compared to guts (~20%)^126,127^, which can lead to the inference of inactive reactions in metabolic models. Despite these current limitations, metabolic modeling has shown promise in pioneering soil studies. Metabolic models have helped elucidate how soil microbes affect soil functions^128,129^, determinants of pathogen invasion success^130^, cross-feeding reactions and design of an herbicide-degrading community^131^, and biostimulant effectiveness^132^. In the future, metabolic models could be leveraged to understand: (1) how ecosystem-level outputs of soils such as greenhouse gas emissions, nutrient cycling, and pollutant degradation depend on the biotic and abiotic context of the soil; (2) how these outputs can be steered using interventions; and (3) through what mechanisms intervention effectiveness depends on the environment and the resident microbiome.

## Future perspectives

Comparing the soil and gut systems led to three important takeaways: (1) we need a systemic shift towards less intensive management practices (healthy soil lifestyle) that increase the diversity of microbes and microbial functions to promote robust soil multifunctionality; (2) innovative solutions such as synbiotics and phage-based interventions may enable effective steering of soil microbiome composition and function; and (3) developing biology-based soil types, machine learning models predicting intervention effects from soil context, and metabolic models may lead to a “personalized” or field-specific management strategy for soils. Nevertheless, the transfer of concepts from the human gut to soil will require contextualization and refinement. The structure of the soil habitat is much more complex than that of the human gut, and current datasets do not capture the full biological complexity of soil microbiomes at the micrometer scale, limiting mechanistic understanding of field-level patterns. For a thorough mechanistic understanding, it is imperative that we study soil microbiomes and interactions across scales, ranging from soil aggregates^11,133^ to controlled mesocosms, fields, landscapes, and the global scale^134,135^. Furthermore, soil microbes interact with diverse fauna, plants, and a wide range of fungi, including mycorrhizal fungi, and these interactions impact soil functioning^2,136–139^. Gut microbes, on the other hand, have far fewer interkingdom interactions. These interkingdom interactions in soils also present additional levers for microbiome steering; for example, crop species and varieties, crop diversity, and crop rotation have strong effects on the soil microbiome and its functions^140,141^. Thus, while gut microbiology highlights exciting avenues for improving soil functioning, the complex nature of soil also indicates that new challenges and opportunities will continue to emerge as we learn to steer soil microbiomes.

A commitment to consistent, sustainable land-management of agricultural soils will be the cornerstone of meeting the European goal of 100% healthy soils by 2050^142^. Additional targeted interventions, such as synbiotics and phage rehabilitation, may be needed to restore soil biodiversity in intensively managed soils^7^. Yet, to effectively implement these targeted interventions, we need to better understand how synbiotics affect the engraftment success of soil inoculants and assess the safety and environmental impacts of phage application in soil systems^143,144^. Moreover, soil synbiotics and phage rehabilitation may even be complementary when applied together, adding completely new soil biodiversity restoration approaches to the existing arsenal. Finally, novel data science approaches such as machine learning and metabolic models will play a crucial role in designing personalized soil management strategies to steer the soil microbiome towards enhanced soil multifunctionality. The eventual promise of such models is the development of digital tools in which farmers can enter soil measurements and management goals and obtain recommendations for effective field-specific management interventions. Successful implementation of such data-driven approaches relies on comprehensive soil biodiversity databases, which require large-scale experimental and observational soil surveys and proper data standardization. In this context, legislation such as the EU Soil Monitoring Law will be critical. Such collaborative efforts will enable us to move towards more multifunctional soils while ensuring sufficient food production.

## Data Availability

No datasets were generated or analyzed during the current study.
